# Cognitive tasks could be biased towards generalists: a lesson from wild non-eusocial bees

**DOI:** 10.1093/beheco/araf054

**Published:** 2025-05-25

**Authors:** Tovah Kashetsky, Nigel E Raine, Jessica R K Forrest

**Affiliations:** University of Ottawa, Department of Biology, 30 Marie Curie Private, Ottawa, Ontario K1N 6N5, Canada; University of Guelph, School of Environmental Sciences, 50 Stone Road EastGuelph, Ontario N1G 2W1, Canada; University of Ottawa, Department of Biology, 30 Marie Curie Private, Ottawa, Ontario K1N 6N5, Canada

**Keywords:** animal cognition, associative learning, diet breadth, exploration, insect pollinators, non-eusocial bees

## Abstract

Ecological niches are closely intertwined with cognition in many animal lineages. For example, diet breadth is linked with performance on tasks measuring learning and exploration in several vertebrates, with generalists often exhibiting faster learning and more exploratory behavior than specialists. We compared associative learning performance and exploratory tendencies between dietary specialist and generalist bee (Anthophila) species using a closed-environment task with free-moving bees called the free-moving proboscis-extension response (FMPER). We found lower participation rates than expected, especially among specialist species, which hindered our ability to answer our primary question. Because participation rates of specialist species were so low, we combined our data with another published dataset that reported results from the same learning task but for several different bee species (again including specialists and generalists) to investigate the relation of diet breadth with associative learning and exploration across a broader species assemblage. Phylogeny-informed generalized linear mixed models indicate that neither specialists nor generalists increased accuracy throughout the task, although bees of both diet breadths became faster at drinking from the rewarding strip. Bees decreased their drinking latency—a measure of exploration—throughout the experiment, with no effect of diet breadth. However, specialists became less likely to participate over the course of the task compared to generalists. Our results suggest that specialist and generalist bees have experienced similar selection for associative learning abilities, and that specialists are hesitant to continue interacting with novel stimuli. Our study highlights the importance of developing cognitive tasks that measure abilities equally across the full range of life history traits.

## Teaser

Bee foraging habits are associated with engagement in cognitive experiments. We compared the cognitive traits of wild bee species that visit many flower types (generalists) with those that prefer specific plants (specialists). While both groups learned equally well, specialists were more reluctant to engage with unfamiliar objects. This suggests that a bee's foraging strategy influences its approach to novel situations, which has important implications for comparative cognitive research.

## Introduction

Environmental pressures can shape cognition by forcing animals to adapt; conversely, expansion of certain cognitive abilities can create access to new niches. Even among closely related species, variation in ecological niches can be reflected in cognitive processes such as spatial memory, exploration, or associative learning (Mettke-Hofmann et al. 2002; Shettleworth 2009; Henke-von der Malsburg et al. 2020). For example, certain bird species such as black-capped chickadees (*Parus atricapillus*) and acorn woodpeckers (*Melanerpes formicivorus*) accumulate food by storing it in their environment for later consumption—sometimes caching hundreds of food items a day and retrieving them months later (Sherry 1985; Healy and Hurly 2004). When tested on spatial memory tasks, these food-caching birds demonstrate superior accuracy and retention compared to closely related non-caching species (Shettleworth et al. 1990). One of the major components of an animal’s ecological niche, dietary breadth, is linked with cognitive performance in many vertebrates. Commonly, vertebrates with broader diets are more exploratory (Tebbich et al. 2009), less fearful of novel stimuli (Bergman and Kitchen 2009), and faster at learning associations (Overington et al. 2011) compared to species with narrower diets.

Approximately a quarter of bee species (Anthophila) in North America are dietary specialists that collect pollen from specific host plant genera or families, while the remainder are generalists that collect pollen from a wider range of plant taxa (Fowler and Droege 2020). We might expect generalists to have superior learning abilities and consequently larger brains than specialists because generalists must learn a greater number of associations between floral cues and rewards, not to mention the handling techniques required for different floral morphologies (Laverty and Plowright 1988; Chittka and Thomson 1997). In contrast, specialist species feed from only their host plant and might therefore rely more heavily on innate preferences. However, a recent study found that specialist bees have, on average, larger brains relative to their body size compared to generalist species (Sayol et al. 2020). Perhaps the relatively larger brain size reflects that specialist bees—unlike generalists—require the cognitive abilities to distinguish the floral characteristics of their host-plants to avoid wasting energy visiting non-host plants (Chittka and Raine 2006). On the other hand, relative brain size might not be a good proxy for learning ability (Chittka and Niven 2009). In a recent study, Collado et al. (2021) found that bee species with larger absolute brain size demonstrated superior performance in an associative learning task compared to species with smaller brains. However, the relationship for relative brain size was less conclusive (Collado et al. 2021). Taken together, results from these studies leave it somewhat unclear whether generalist or specialist bee species have superior associative learning abilities or cognitive traits more broadly. Testing for a link between dietary breadth and learning would allow us to understand how ecological niches and cognition might be connected in bees.

In this study, we tested for differences in the associative learning abilities and exploratory tendencies between dietary specialist and generalist bee species, while accounting for phylogenetic relatedness. We expected specialists and generalists to differ in their cognitive abilities. On one hand, specialists could have better associative learning skills, allowing them to better discriminate their host plants from other taxa. Alternatively, selection could have favored better associative learning abilities in generalist bees compared to specialists, as generalists must learn associations between rewards and floral cues from multiple flower species (Raine et al. 2006). If the latter scenario is correct, the discrepancy in relative brain size between specialist and generalist species (Sayol et al. 2020) could stem from other perceptual or cognitive differences related to their dietary niches. Further, we expected specialists to be less exploratory than generalists, as specialists interact with their familiar host-plants while generalists forage from a wider variety of plants and thus could be accustomed to interacting with more novel stimuli.

We tested various specialist and generalist species of non-eusocial bees on an associative learning task, the free-moving proboscis-extension response (FMPER). FMPER was adapted from the original classical conditioning protocol called the proboscis-extension response (PER) designed by Takeda (1961) and perfected by Bitterman et al. (1983), which Muth et al. (2018) modified to allow testing of wild bees directly in the field. However, while conducting our research, we observed a lower participation rate than expected, especially among specialist species. In order to answer our question about cognitive differences between specialists and generalists, we therefore merged our data with those from Collado et al. (2021), the only other published study to have used FMPER on non-eusocial specialist bees. Collado et al. (2021) measured the relation between brain size and learning (using FMPER) without considering dietary breadth; however, their dataset included both specialist and generalist bee species. Thus, combining our data with theirs allows us to answer our question about the relationship between learning and diet breadth, while asking a novel scientific question not explored with their data. In addition to measuring associative learning performance, we compared exploratory tendencies between specialist and generalist species. Our study highlights the importance of considering how life history traits can bias measures of cognitive performance and reduce the generalizability of experimental findings.

## Methods

### Experimental overview

Between May and August of 2022, we conducted our experiment in green spaces and farms around Ottawa, Canada, with necessary permissions. Approval from an ethics committee was not required, but we treated bees with respect, assuming they experience emotion and pain. We studied female non-eusocial bees, excluding males because only females collect pollen for their offspring, and only pollen collection (not nectar) is relevant to dietary breadth in bees (Wcislo and Cane 1996).

Experiments were conducted in the field under an insect tent (L × W × H: 3.0 × 3.0 × 2.1 m). After catching bees on wildflowers, we held them in individual cylindrical polyethylene vials (L × H: 50 × 25 mm) inside a cooler around 17°C for 45 to 175 min to promote hunger and motivation to participate in the food-based learning task. Fifteen minutes prior to beginning an experimental trial, we removed the bee from the cooler and placed her on the experimental table for acclimation, keeping her in the shade throughout the experiment.

### Learning assay

We used a modified version of the free-moving proboscis-extension response (FMPER) assay (Muth et al. 2018), which is an associative learning paradigm that has successfully been used with wild-caught eusocial (Muth et al. 2018) and non-eusocial bees (Collado et al. 2021). The FMPER assay tests whether an individual bee can learn an association between a color and a reward. In our study, the FMPER assay consisted of seven learning trials before a final unrewarded test.

**Figure 1.**
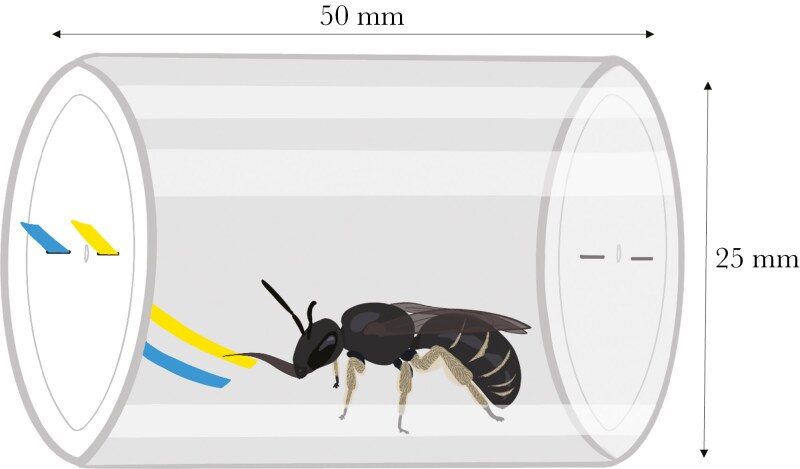
Schematic illustration of a bee in an FMPER experimental vial drinking from one of the two paper strips (blue and yellow), in which one color is dipped in a rewarding sucrose solution and the other is dipped in an aversive salt solution. Illustrated by T. Kashetsky.

Each learning trial began with the insertion of two paper strips (L × W: 23 × 3 mm; acid-free Recollection smooth cardstock), one yellow (MP267780) and one blue (MP470530), into the vial (Fig. 1). The reflectance spectra from these colored paper strips were plotted in a bee color space (Figure S1) to ensure bees would be able to perceive them differently and discriminate between them. Experimental vials had two rectangular holes in each end, allowing us to insert the strips in the end furthest from the bee. A white cap covered one end of the vial while the other was painted white on the outside, ensuring the bee experienced a consistent background regardless of the end through which the strips were inserted. One strip was dipped in a rewarding solution (50% sucrose solution w/w), while the other strip was dipped in an aversive solution (5% NaCl solution w/w), and both solutions were made fresh every other day and kept refrigerated (Muth et al. 2018). Although some bees are commonly observed consuming sweat, such as sweat bees (Halictidae), the 5% salt concentration we used was more than 55 times the average sodium concentration of human sweat (0.09% according to Montain et al. 2007), and high NaCl concentrations are known to be aversive to honeybees (von Frisch 1934) and bumblebees (Muth et al. 2018). The rewarding color was randomized among and consistent within bees, and fresh strips were used each trial. The rewarding strip was placed alternately on the left or the right side of the vial, relative to the bee. Vials were rotated between trials so that each slot only ever had contact with one solution (and water during the test) to avoid contamination between strips. For example, if the slot for the rewarding strip was on the right, then we would rotate the vial 180 degrees for the next trial, so the same slot would now be on the left. Using modeling clay, we crafted a holder for the vial to prevent it from rolling on the table, along with a holder for the paper strips to keep them in a consistent and uniform position inside the vial for each trial to avoid accidentally touching a strip to a bee before she approached.

Once each learning trial began, we recorded the time that the bee first extended her proboscis to one of the strips, and we removed that strip after allowing her to drink for 3 to 5 s. The bee then had 2 min to drink from the remaining strip before it was removed, or it was removed 3 to 5 s after the bee drank from it. The trial was considered successful if the bee drank from the rewarding strip, regardless of whether she also drank from the aversive strip. The trial was considered a failure if the bee only drank from the aversive strip. We also recorded the order of strips that the bee drank from. If the bee did not drink from either strip within 15 min, we excluded the individual from further trials and did not administer the test. There was a 2-min rest interval between trials. After seven of these rewarded training trials, we tested the bee using the same protocol, except both strips were dipped in water (the unrewarded “test”). Bees were then brought back to the laboratory and placed in the freezer for later identification.

The learning assay protocol followed by Collado et al. (2021) can be found in their published article. There were several differences in methodology between the current study and that of Collado et al. (2021), most of which we could account for prior to merging datasets. A notable difference between the datasets is that bees were tested throughout the eight FMPER trials even if they did not participate in a trial in the Collado et al. (2021) dataset, while in our study, we ceased training once individuals did not participate. Several other minor differences in the experimental protocols between these studies (e.g., trial duration, non-rewarding stimuli) are outlined in the supplementary information.

### Behavioral measures

Our initial interest was quantifying success in the test phase and the training trials. Success on the test was defined as drinking from the color strip associated with the reward during the preceding learning trials. Success throughout the learning trials was measured with two metrics: sucrose latency, defined as the time it took the bee to drink from the rewarding strip during each trial; and correct first choice, defined as the bee drinking from the rewarding strip first, regardless of whether she then drank from the aversive strip or not. Once we encountered the poor participation rates, we discarded the test success measure, as we had insufficient sample size to analyze it effectively. We then created two additional measures that may reflect exploration of novel objects (Muller et al. 2010): drinking latency, a continuous variable defined as time until the bee drank from either strip, and participation, a binary variable defined as whether or not each individual bee sampled either strip within each trial.

### Identification and traits

Bees tested in the current study were initially identified to genus using Carril and Wilson (2023) and Discoverlife.org (Ascher and Pickering 2010). If the genus included local dietary specialists and generalists, specimens were identified to species using Sheffield et al. (2011) for *Megachile*, Gibbs et al. (2014) for *Dufourea*, Mitchell (1960) for *Andrena*, *Protandrena*, *Macropis*, and *Colletes*, and Mitchell (1962) for *Xenoglossa*, *Melissodes* and *Anthophora*. If the genus included no dietary specialists in our study region, genus-level identification was sufficient for creating our phylogenetic tree, with the exception of *Lasioglossum*. To properly incorporate our *Lasioglossum* specimens into the phylogenetic tree with the two *Lasioglossum* (*Sphecodogastra*) species from Collado et al. (2021), we identified our *Lasioglossum* to subgenus. None of our specimens were of the subgenus *Sphecodogastra*; thus, we used exemplar species for our subgenera. See the supplementary material for a list of species used in the phylogeny (Table S1). Taxonomic information about the bees from Collado et al. (2021) was taken from that paper’s supplementary materials.

Dietary breadth was assigned to bee species using sources outlined in Table S1, with the aid of Stuart Roberts for species from Collado et al. (2021). Species classified as dietary specialists collect pollen from one host genus or family, while generalists collect pollen across several families. *Megachile campanulae* can be considered a generalist with a preference for *Campanula* or a specialist. Our results remained qualitatively unchanged with *M. campanulae* coded with either diet breadth; thus, we coded *M. campanulae* as a generalist. Because we expected that body size might affect participation in the learning task (i.e., smaller bees might reach satiation sooner than larger bees), we also obtained measures of inter-tegular distance (ITD; the distance between the tegulae—a well-established proxy of body size for bees (Cane 1987)) for all individuals in this study. We measured ITDs for the specimens in our dataset and used the measurements reported by Collado et al. (2021) for the specimens in their dataset. The specimens collected for this study, not including those of Collado et al. (2021), are housed in the Forrest Lab collection at the University of Ottawa with vouchers deposited at the Canadian National Collection of Insects, Arachnids, and Nematodes in Ottawa, Canada.

### Analyses

#### Combining datasets.

Prior to merging datasets, we excluded data from males and parasitic species from the Collado et al. (2021) dataset because we were only concerned with bees that collect pollen. Additionally, we excluded eusocial species from the Collado et al. (2021) dataset because we only tested non-eusocial bees. Finally, we excluded individuals from their dataset that were identified only to genus if we could not confidently assign a diet breadth to them. In total, we used 82 individuals from their dataset of 204 bees.

For our model testing for differences in sucrose latency, we scaled the sucrose latency variable within each dataset to account for the different trial durations, using the scale() function from the standardize package (Eager 2022) to center each set of latency values around zero. In our model testing for differences in drinking latency, bees that did not participate in a trial were assigned the maximum number of seconds in the trial. Thus, in the Collado et al. (2021) dataset, non-participating bees were assigned 120 s, while in the current study, non-participating bees were assigned 900 s. Because we used matrices for correct first choice and drinking latency (explained below in the *Statistical Analysis* section) and participation was binary, scaling these three variables was not required.

#### Phylogeny.

To account for relatedness in our statistical models, we incorporated a phylogenetic covariance matrix derived from a phylogenetic tree including the species in the current study and those studied by Collado et al. (2021). We used a tool for building phylogenetic trees for bee species called BeeTree (Henríquez-Piskulich et al. 2024). We chose a common species found in the sampling area as a stand-in for the eight taxa that were identified to genus level, and five stand-in species for the five *Lasioglossum* subgenera. This software contained 46 of the 53 taxa required for our tree. For the remaining seven species, we used a closely related species as a substitute in the phylogenetic tree. See the supplementary material for a list of species used in the phylogeny (Table S1) and the phylogenetic tree (Figure S2).

#### Statistical analysis.

We organized the data using the tidyverse package (Wickham et al. 2019) in R version 4.1.2 (R Core Team 2021). We converted our non-ultrametric phylogenetic tree into an ultrametric tree using the chronoMPL() function from the package ape, version 5.8 (Paradis et al. 2004). We then created an inverse covariance matrix using the inverseA() function from the MCMCglmm package, version 2.36 (Hadfield 2024). To analyze our behavioral measures, we used four phylogeny-informed generalized linear mixed models (GLMM) with the MCMCglmm() function from the MCMCglmm package with the burn-in set to 3,000, thinning interval set to 10, and total MCMC intervals set to 100,000. For all four models, taxon and individual bee ID were included as random effects, the inverse covariance matrix was incorporated into the model, and we assessed fit for all four models with the plot() function and examined the model outputs with the summary() function using base R functions.

The first model, quantifying associative learning performance throughout the training phase of FMPER and the unrewarded test, was a Gaussian GLMM containing the scaled sucrose latency value as the dependent variable, modeled as a function of trial number (coded as a continuous variable), diet breadth, an interaction between trial number and diet breadth, the color of the rewarding paper strip, and the study dataset as fixed effects.

Our second model was a binomial GLMM used to test whether the proportion of correct first choices throughout the training phase and unrewarded test differed between dietary specialists and generalist taxa. Each individual was assigned a binary value of 1 for drinking from the rewarding stimulus as their first or only choice, or 0 for drinking first from the non-rewarding stimulus. We modeled the binary value of each individual's first choice as a function of trial number, diet breadth, an interaction between trial number and diet breadth, the color of the rewarding paper strip, and the study dataset as fixed effects. The family was specified as “categorical,” which is equivalent to binomial for MCMCglmm().

We initially planned to compare learning in the unrewarded test between diet breadths with a GLMM; however, because only three specialist individuals participated in the test while 51 generalist individuals participated, we were unable to perform a GLMM to model success for the test. Instead, we ran models to quantify our additional behavioral measures: drinking latency and participation. We used a truncated Gaussian GLMM (Burkardt 2014) as our third model to test drinking latency because our data were approximately normally distributed, but truncated at the maximum time allowed for drinking (i.e., trial duration). To accommodate the trials of individuals that did not participate, we fitted drinking latency to a two-column response matrix as the dependent variable using the cbind() function, consisting of a column with the minimum value of an individual’s drinking latency and another column with the maximum value of that individual’s drinking latency. If an individual bee participated in the trial, both the minimum and maximum drinking latency columns contained the same value: the number of seconds it took them to drink from the first strip. If an individual bee did not participate, they were assigned the maximum number of seconds per trial (900s for the current study, 120s for Collado et al. (2021)) for the minimum drinking latency score, and an infinity value for the maximum drinking latency score. The drinking latency matrix was modeled as a function of trial number, diet breadth, an interaction between trial number and diet breadth, the color of the rewarding paper strip, and the study dataset as fixed effects. The family was specified as “cengaussian,” which was used to truncate the distribution.

Our final model was a binomial GLMM to test if participation rates differed between dietary specialists and generalist taxa. A binary value for each individual at every trial was the dependent variable, with 1 for participation and 0 for nonparticipation, even if bees were not tested in the trial due to lack of participation. Participation was modeled as a function of diet breadth, trial number, an interaction between diet breadth and trial number, the color of the rewarding paper strip, and the study dataset as fixed effects. We included ITD as an additional covariate after visual inspection of the data confirmed there was no collinearity between diet breadth and ITD. The family was specified as “categorical” to create a binomial distribution using MCMCglmm().

## Results

We analyzed data from 158 individuals tested in the current study (referred to as the Kashetsky et al. (2025) dataset) in addition to the 82 individuals from the Collado et al. (2021) dataset, for a total of 240 bees in the combined dataset. Eighty-six individuals were specialists from 14 taxa (mean number of individuals per taxon ± SD, 6.1 ± 6.0), while 154 individuals were generalists from 39 taxa (mean number of individuals per taxon ± SD, 3.9 ± 5.2). Of the 240 individuals tested, 123 bees participated in at least one trial, and 20 of these participated in all eight trials (including the final unrewarded test trial).

**Figure 2.**
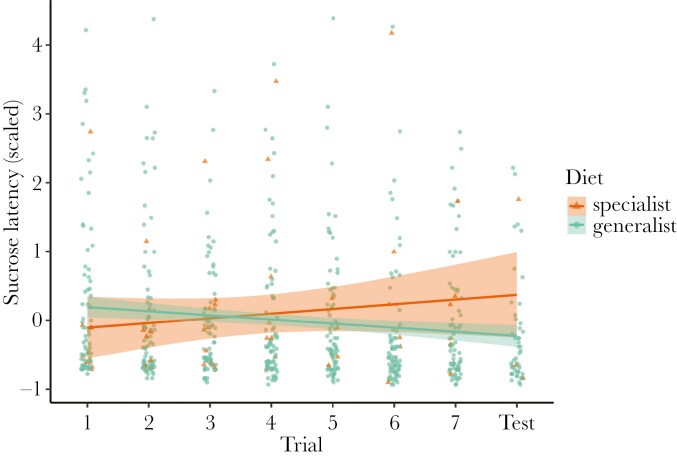
Sucrose latency (scaled to the mean sucrose latency for each dataset and expressed in standard deviation units) throughout the seven training trials and unrewarded test for dietary specialists (n = 18) and generalists (n = 103). Each data point is an individual bee’s sucrose latency for each trial they participated in. The lines show the predicted values from the geom_smooth() function from ggplot, while the shading shows the 95% confidence intervals.

Sucrose latency decreased over the course of the trials (Table 1). This decrease appears to be mainly driven by generalists, as specialists showed no clear temporal trend in latency (Fig. 2). However, there was no significant interaction between diet breadth and trial number, nor was there a significant overall difference in latency between specialists and generalists. We did not detect any effect of the color of the rewarding stimulus or study dataset. Taxon had a moderate effect on sucrose latency (Figure S3) and individual ID had a small effect (Table 1).

**Table 1.** Results of the phylogenetically informed generalized linear mixed model comparing sucrose latency between dietary specialists (n = 18) and generalists (n = 103). Fixed effects with pMCMC < 0.05 and 95% CIs that do not overlap zero, and random effects with 95% CIs that do not overlap zero, are shown in bold.

	Posterior mean	95% CI (lower)	95% CI (upper)	pMCMC
***Fixed effects***				
Intercept	0.45	−0.01	0.94	0.046
**Trial**	**−0.05**	**−0.09**	**−0.01**	**0.007**
Diet: specialist	−0.16	−0.93	0.57	0.67
Color of correct stimulus: yellow	−0.17	−0.37	0.01	0.08
**Dataset: Kashetsky et al. (2025)**	−0.17	−0.54	0.16	0.30
Trial * Diet: specialist	0.04	−0.11	0.20	0.57
***Random effects***				
**Taxon**	**0.17**	**0.003**	**0.50**	
**Individual ID**	**0.07**	**0.003**	**0.14**	

**Figure 3.**
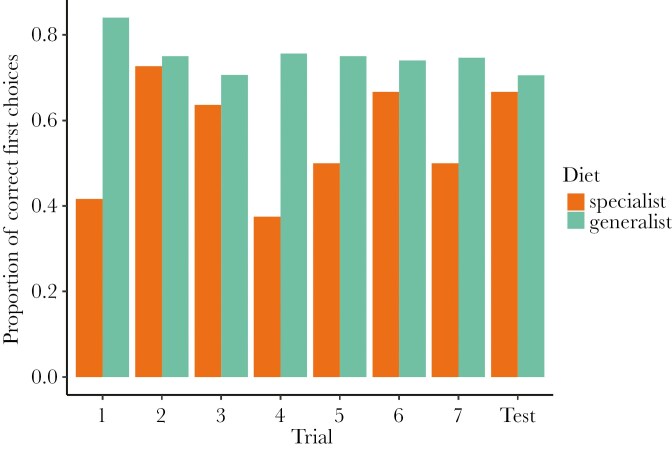
The proportion of bees with correct first choices throughout the seven training trials and unrewarded test from the number of dietary specialist (n = 20) and generalist (n = 103) individuals that participated. To calculate the proportion of correct first choices, the number of bees within a trial that drank only from the rewarding stimulus or from the rewarding stimulus before the aversive/non-rewarding strip was divided by the total number of bees that participated within a trial.

We detected no effect of trial, diet breadth, rewarding stimulus color, nor an interaction between trial number and diet breadth on the proportion of correct first choices (Fig. 3; Table 2). There was a significant effect of study dataset, with the current study having higher rates of correct first choices. Taxon and individual ID each had a small effect on the proportion of correct first choices (Table 2).

**Table 2.** Results of the phylogenetically informed generalized linear mixed model comparing the proportion of bees with correct first choices between dietary specialists (n = 20) and generalists (n = 103). Fixed effects with pMCMC < 0.05 and 95% CIs that do not overlap zero, and random effects with 95% CIs that do not overlap zero, are shown in bold.

	Posterior mean	95% CI (lower)	95% CI (upper)	pMCMC
***Fixed effects***				
**Intercept**	**3.35**	**-11.70**	**17.50**	**0.56**
Trial	0.44	−0.45	1.55	0.33
Diet: specialist	−12.32	−36.36	4.50	0.15
Color of correct stimulus: yellow	1.99	−2.69	7.17	0.39
**Dataset: Kashetsky et al. (2025)**	**12.33**	**0.60**	**25.93**	**0.01**
Trial * Diet: specialist	1.02	−2.24	5.04	0.54
***Random effects***				
**Taxon**	**169.96**	**0.04**	**507.89**	
**ID**	**28.15**	**0.002**	**97.49**	

**Figure 4.**
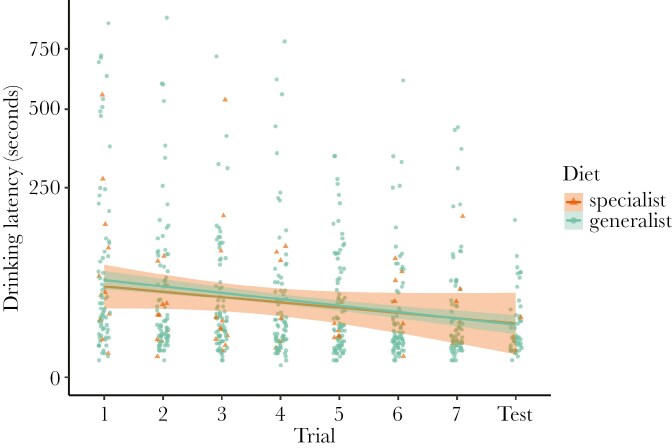
Drinking latency (number of seconds until a bee drank from either strip) throughout the seven training trials and unrewarded test for dietary specialists (n = 20) and generalists (n = 103). Each data point is an individual bee’s drinking latency for each trial in which they participated. The lines show the predicted values from the geom_smooth() function from ggplot, while the shading shows the 95% confidence intervals. Note square-root scaling of the y-axis for visualization.

Drinking latency decreased over the eight trials and was not different between dietary specialists and generalists (Fig. 4; Table 3). We detected no interaction between trial number and diet breadth, nor an effect of the rewarding stimulus color. Study dataset had a significant effect on drinking latency, with bees in the current study taking longer to drink, which is in accordance with the longer trial periods in the current study (Table 3). Taxon had a large effect on drinking latency while individual ID had a moderate effect (Table 3).

**Table 3.** Results of the phylogenetically informed generalized linear mixed model comparing drinking latency between dietary specialists (n = 20) and generalists (n = 103). Fixed effects with pMCMC < 0.05 and 95% CIs that do not overlap zero, and random effects with 95% CIs that do not overlap zero, are shown in bold.

	Posterior mean	95% CI (lower)	95% CI (upper)	pMCMC
***Fixed effects***				
Intercept	160.48	−184.04	501.59	0.33
**Trial**	**−9.00**	**−11.34**	**−6.61**	**0.0001**
Diet: specialist	177.94	−56.75	398.28	0.12
Color of correct stimulus: yellow	6.48	−25.98	41.41	0.72
**Dataset: Kashetsky et al. (2025)**	**591.04**	**395.21**	**808.01**	**0.0001**
Trial * Diet: specialist	0.02	−7.16	7.17	0.98
***Random effects***				
**Taxon**	**200089.08**	**56513.42**	**413254.30**	
**ID**	**3344.98**	**1740.43**	**5244.61**	

**Figure 5.**
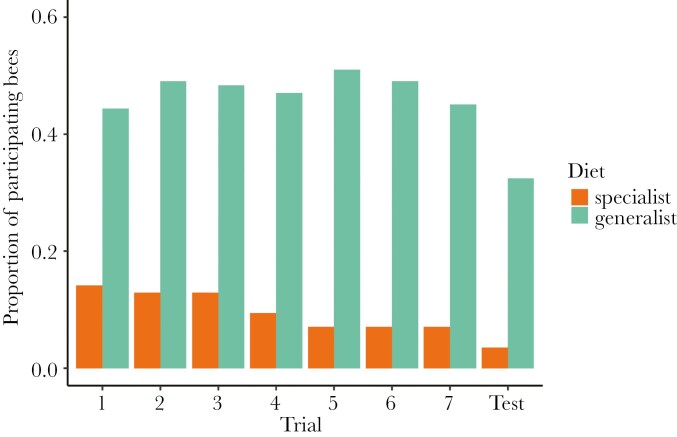
The proportion of bees that participated throughout the seven training trials and unrewarded test from the total number of individuals tested for dietary specialists (n = 85) and generalists (n = 151). One specialist and three generalists were excluded for missing ITD measurements. To calculate the proportion of participating bees, we divided the number of bees that participated in that trial by the total number of specialist or generalist bees tested in the experiment.

**Figure 6.**
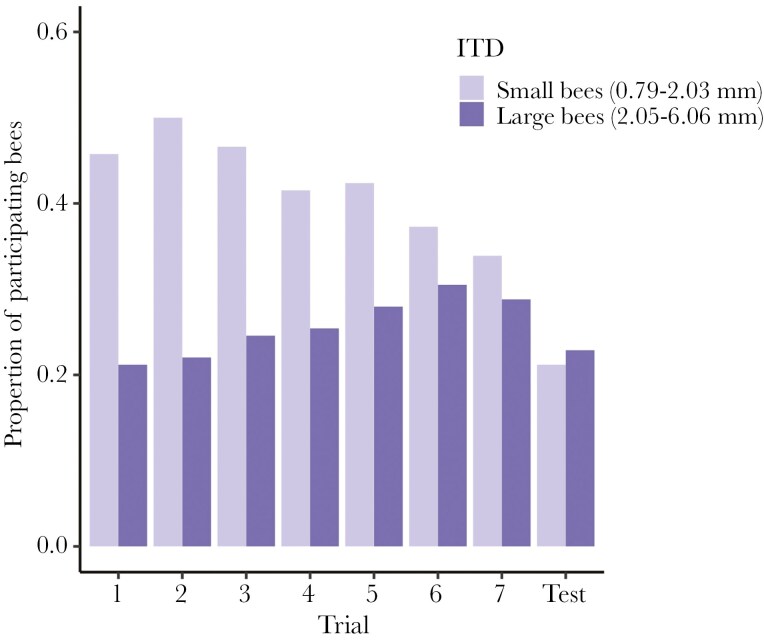
The proportion of bees that participated throughout the seven training trials and unrewarded test from the total number of individuals tested, categorized as small (n = 118) or large bees (n = 118) for the purpose of this figure; four bees were excluded for missing ITD measurements. To calculate the proportion of participating bees, we divided the number of bees that participated in that trial by the total number of small or large bees tested in the experiment. Dietary specialists and generalists are grouped together for this figure.

Participation decreased over the trials for both specialists and generalists (Table 4). We did not detect an overall difference in participation between diet breadths; however, we detected a significant interaction between diet breath and trial that indicates specialists were less likely to participate as the trials progressed compared to generalists (Fig. 5; Table 4). Further, we detected a significant decrease in participation with increasing ITD and a positive interaction between ITD and trial, meaning that larger bees were less likely to participate than smaller bees, but increased their participation relative to smaller bees throughout the task (Fig. 6; Table 4). This effect of body size could be driven in part by *Lasioglossum*, which are small (mean ITD ± SD, 1.57 ± 0.34 mm) and had a high participation rate (49 out of 53 individuals tested participated in at least one trial). There was an effect of study dataset on participation, with the current study having a lower participation rate than Collado et al. (2021), perhaps because Collado et al. (2021) ceased testing taxa that did not participate. We detected no effect of the rewarding stimulus color. We found a large effect of taxon on participation and a moderate effect of individual ID (Table 4).

**Table 4.** Results of the phylogenetically informed generalized linear mixed model comparing participation between dietary specialists and generalists (n = 85 and 151 respectively; one specialist and three generalists were excluded for missing ITD measurements). Fixed effects with pMCMC < 0.05 and 95% CIs that do not overlap zero, and random effects with 95% CIs that do not overlap zero, are shown in bold.

	Posterior mean	95% CI (lower)	95% CI (upper)	pMCMC
***Fixed effects***				
**Intercept**	**7.05**	**2.61**	**11.77**	**0.003**
Diet: specialist	−1.20	−3.42	1.27	0.31
**Trial**	**−0.94**	**−1.17**	**−0.69**	**0.0001**
**ITD**	**−2.80**	**−4.18**	**−1.42**	**0.0001**
Color of correct stimulus: yellow	−0.52	−1.48	0.38	0.28
**Dataset: Kashetsky et al. (2025)**	**−3.21**	**−5.19**	**−1.50**	**0.0002**
Diet: specialist * Trial	**−0.36**	**−0.57**	**−0.16**	**0.0001**
Trial * ITD	**0.40**	**0.29**	**0.51**	**0.0001**
***Random effects***				
**Taxon**	**13.32**	**2.51**	**26.11**	
**ID**	**8.19**	**4.80**	**11.96**	

## Discussion

In this study, we tested for a relationship between diet breath and two cognitive traits in wild non-eusocial bees using FMPER, an associative learning task created for testing wild bees (Muth et al. 2018). Our hypothesis that cognition differs between specialist and generalist bees is partially supported, although our findings have different implications than anticipated. We expected that cognitive differences stemming from foraging behavior would present themselves through variation in associative learning performance, reflected in sucrose latency and the proportion of bees with correct first choices, and exploratory tendencies, reflected in drinking latency or participation. Instead, we found that sucrose latency (Fig. 2; Table 1) decreased throughout the eight trials and did not differ between specialist and generalist taxa, while we did not detect an effect of trial number or diet breadth on the proportion of bees with correct first choices throughout the experiment (Fig. 3; Table 2). Although bees were becoming faster throughout the task, they were not improving accuracy, and diet breadth did not impact either of these associative learning measures. We detected a significant effect of study dataset on the proportion of correct first choices, with individuals in the current study outperforming individuals in Collado et al. (2021). Perhaps the difference in our experimental protocol led to improved performance: we continued administering the task only with participating individuals, while Collado et al. (2021) continued the experiment even when bees did not participate. Drinking latency decreased throughout the eight trials and did not differ between specialist and generalist taxa (Fig. 4; Table 3). Participation decreased for both diet breadth groups throughout the trials, while a signification interaction between diet breadth and trial number revealed that specialists were less likely to continue participating throughout the trials than generalists (Fig. 5; Table 4). Accordingly, dietary breadth predicts the willingness to continue interacting with the stimuli throughout the task, but not the speed at which the interaction occurs. Smaller bees were more likely start participating compared to larger bees, while larger bees increased their participation throughout the trials (Fig. 6; Table 4), likely a reflection of variation in hunger.

### Cognitive differences between dietary specialists and generalists

The lack of detectable difference in learning performance between specialists and generalists could in part be due to the lack of participation from specialists leading to low statistical power. Alternatively, the absence of a relationship between diet breadth and associative learning performance could be real: throughout evolutionary history, specialist and generalist bees might have experienced similar selection on their associative learning capabilities. Perhaps the foraging behaviors of specialist bees still require some level of associative learning (Strickler 1979; Laverty and Plowright 1988). Conversely, generalist bees might rely less heavily on learning associations between floral cues and rewards, instead foraging more opportunistically. Our results, in conjunction with the ambiguous relationship between relative brain size and associative learning performance documented by Collado et al. (2021), suggest that the larger relative brain sizes of specialist bees (Sayol et al. 2020) could reflect cognitive functions other than associative learning. Cognitive abilities such as discriminating amongst flowers or navigating to host-plants might be the driving factors behind the larger relative brain sizes of specialists. Indeed, mushroom bodies—the neural region crucial for learning and memory—increase in volume with foraging and thus navigation experience in the solitary alkali bee (*Nomia melanderi*) (Hagadorn et al. 2021) and the eusocial honeybee (*Apis mellifera*) (Fahrbach et al. 1998).

Paradoxically, diet breadth predicted participation throughout the trials, but not drinking latency—even though both measures are indicators of exploration. Our binary measure of participation captured whether an individual took part in a trial, while our continuous variable of drinking latency measured the time an individual took to approach and drink from a strip. Our findings suggest that specialist and generalist bee taxa differ in their tendency to engage with stimuli throughout the trials, but when they do explore novel stimuli, they do so at a similar speed. Comparatively lower participation rates in specialist bees indicate that they are more selective about the stimuli with which they interact (in this case drinking from the paper strips), which could be related to their preferences for foraging only from their host-plants. In turn, generalist bees collect pollen from a wider variety of plants than specialists, and thus interact with more floral colors, odors, and morphologies, which could explain generalists’ greater willingness to interact with the FMPER stimuli.

### Implications for cognitive testing

Historically, studying animal cognition has primarily been accomplished through laboratory research with domesticated or commercialized animals. Although this research has led to important discoveries (e.g., operant conditioning in pigeons (Skinner 1938); spatial learning in rats (O’Keefe and Dostrovsky 1971)), laboratory testing restricts researchers to studying animals that behave relatively normally in captivity. This criterion not only limits the scope of cognitive studies, it also hinders the generalizability of cognitive research to wild animals (Lihoreau et al. 2019). The growing field of studying cognitive ecology in the wild requires carefully crafted tasks that wild animals will participate in, and that also measure the intended cognitive processes (Buchanan et al. 2013; Morand-Ferron et al. 2016). FMPER is a novel tool for studying learning in wild bees and is more flexible than the standard PER protocol, allowing researchers to expand cognitive research to non-eusocial bees which are notoriously difficult to keep in captivity. However, a shortcoming of FMPER, or any experiment using wild animals, is the inability to control for experience prior to participation in the task. Regardless, studying cognition with wild-caught animals is important for understanding cognitive abilities across a range of animal taxa.

Here, we observed substantial variation among taxa in participation rates, which limits the utility of the task for interspecific comparisons. In particular, because we found that specialist bees are less likely to participate throughout the FMPER trials than generalists, we caution users to consider this bias when designing experiments. Even among generalists, the highest participation rate on any trial was only 52%. Further, we found that body size predicted initial and continued participation in the task in a contradictory manner: small bees were more likely to participate than large bees, but decreased participation throughout the trials, while larger bees were initially less likely to participate, but increased participation throughout the trials. These participation trends could be the result of small bees becoming satiated and large bees requiring a longer waiting periods to promote hunger. FMPER could be an effective protocol to study associative learning when comparing performance within a bee taxon that participates readily in the task; hence, preliminary trials should be completed and researchers could modify the task to improve participation and accuracy.

The FMPER protocol already offers several customizations (Muth et al. 2018). For instance, the task can be conducted with a rewarding stimulus (sucrose solution) contrasted with a non-rewarding stimulus (water) or an aversive stimulus (NaCl solution or quinine). Decision-making accuracy tends to be favored when costs of mistakes are high, while speed is favoured when the cost of errors is low (Chittka et al. 2003; Burns 2005). Our study used an aversive salt solution (5% NaCl), while Collado et al. (2021) used water as a non-rewarding stimulus; thus, the cost of selecting the incorrect stimulus was greater in our study. However, we did not detect an effect of study dataset nor an interaction between study and trial on the proportion of correct first choices, suggesting that our salt solution was also not costly enough to promote accuracy. Although NaCl solution can be aversive to honeybees (von Frisch 1934) and bumblebees (Muth et al. 2018), perhaps it is not as aversive to non-eusocial bees. To our knowledge, quinine solution has yet to be tested with non-eusocial bees, but is aversive to honeybees (Avarguès-Weber et al. 2010) and bumblebees (Chittka et al. 2003), thus holds potential for research with non-eusocial bees. Assessing individuals that engage in the learning trials, thus ensuring they are performing the learning task, and discontinuing the experiment with individuals that do not participate could improve accuracy, as suggested by the higher proportion of individuals in our study making the correct first choice compared to those in Collado et al. (2021) (Table 2). Reducing the number of trials could be another FMPER customization. Because we found specialists were less likely to participate throughout the course of the experiment, and participation decreased more rapidly after the fifth and sixth trials for small and large bees, respectively, it might be favorable to reduce the number of trials. Learning in solitary bees has been demonstrated with as few as two training trials (Collado et al. 2020), thus reducing the number of trials is feasible. There have also been various stimulus adaptations for FMPER, including olfactory stimuli (Amaya-Márquez et al. 2019; Sprayberry 2020) and shapes paired with olfactory stimuli (Jordan et al. 2024). Although olfactory stimuli are more difficult to implement, they have elicited participation in two commercially-available solitary bee species (*Osmia lignaria* and *Megachile rotundata*; Stanley-Stahr and Pitts-Singer 2023) and thus are promising for future research. Additionally, various colors can be used, as long as they are perceptually different to bees (Chittka et al. 1992).

To encourage participation across multiple taxa, researchers might need to compromise on FMPER consistency between studies. For example, specialists might be more inclined to participate if olfactory host-plant cues, or even intact flowers, are used as stimuli. A potential modification could use two host-plant flowers as stimuli, with one maintained as rewarding while the other is modified to be aversive (nectaries drained and filled with a quinine solution), non-rewarding (nectaries drained and filled with water), or less-rewarding (nectaries drained and filled with various concentrations or volumes of nectar). Varying nectar concentration or volumes to differentiate between flower options has been successful in learning tasks with solitary bees (Perez and Waddington 1996; Amaya-Márquez and Wells 2008). Because both flowers appear and smell identical, the location (e.g., left or right side) would be the information that requires learning. In this case, spatial memory would be tested, as opposed to color cues. These major modifications could greatly improve task participation for bees, especially specialists, but at the cost of customizing each apparatus for a specific taxon and having inconsistent stimuli across taxa. If researchers are testing a single taxon or numerous taxa that feed from the same flowers, these two costs become less concerning. Although uniformity across experiments for a task such as FMPER would be ideal for scientific comparison, compromises might be necessary when trialing new cognitive tasks for studying wild animals. Researchers could attempt to use existing protocols other than FMPER to compare learning across solitary bees, such as measuring foraging patterns (Dukas and Real 1991; Orth and Waddington 1997; Bar-Shai et al. 2011; Somanathan et al. 2019).

Tasks that entail a higher cost than foraging errors could promote higher accuracy. In nature, a mistake while foraging for nectar can result in drinking low-quality nectar or landing on a flower with depleted nectaries—both of which are only marginally costly. Pollen foraging errors can be more costly, as collecting pollen from the “incorrect” plant can hinder offspring success in both specialists (Praz et al. 2008) and generalists (Sedivy et al. 2011); however, most mother bees are likely unaware of these costs, as most non-eusocial bees do not partake in offspring care after they have provisioned their nest (Michener 2000). A task relating to nesting behavior (Menzel et al. 1988) or that has major and observable fitness consequences, such as nest parasitism or predation, could elicit higher accuracy, compared to a foraging-based task. For example, Loukola et al. (2020) found that non-eusocial bees can learn to associate a shape with the presence of a brood parasite and subsequently avoid nesting in a cavity with an identical shape. Similarly, Howard (2021) found that a non-eusocial sweat bee (*Lasioglossum lanarium*) avoided approaching colors associated with a predator.

## Conclusion

Contrary to our expectations, specialist and generalist bee species did not differ in their associative learning performance, which could be the result of comparable selection on associative learning abilities regardless of diet breadth. Further, specialists and generalists explored novel stimuli at a similar speed, although specialists abandoned the learning task more rapidly than generalists. This could indicate that specialists are less willing to continue interacting with novel stimuli, a pattern that could be related to their preferences to forage only from their host-plants.

Our findings indicate that there are fundamental differences in the extent to which specialist taxa are willing to engage in activities outside their usual behavioral range. Although cognitive tasks would ideally be applicable for a variety of species, existing methods for studying cognitive abilities likely need to be customized for each taxon to encourage participation, especially for specialists. If cognitive tasks, such as FMPER, can be modified to better suit target species, they have great potential for studying cognitive abilities in wild non-eusocial bees.

## Supplementary Material

araf054_suppl_Supplementary_Figures_S1-S3_Table_S1

## Data Availability

Analyses reported in this article can be reproduced using the data provided by Kashetsky et al. (2025).
