# Supplementary material for: Cognitive tasks could be biased towards generalists: a lesson from wild non-eusocial bees
Source: Behav Ecol. 2025 May 25;36(4):araf054. doi: 10.1093/beheco/araf054 (PMC12302503; doi:10.1093/beheco/araf054)
Supplement: araf054_suppl_Supplementary_Figures_S1-S3_Table_S1 [file araf054_suppl_supplementary_figures_s1-s3_table_s1.docx]

## **Cognitive tasks could be biased towards generalists: a lesson from wild non-eusocial bees**

## Tovah Kashetsky^1*^, Nigel E. Raine^2^, Jessica RK Forrest^1^

^1^University of Ottawa, Department of Biology, Ottawa, Ontario, Canada, K1N 6N5

^2^University of Guelph, School of Environmental Sciences, Guelph, Ontario, Canada, N1G 2W1

*Corresponding author: tovah.kashetsky@uottawa.ca

## **Supplementary material**

### **Differences between the current study and Collado et al. (2021)**

Collado et al. (2021) had trials that lasted a maximum of 2 minutes, followed by 10-minute rest intervals, while we had trials that lasted a maximum of 15 minutes, followed by 2-minute rest intervals. Collado et al. (2021) used water as a non-rewarding stimulus, while we used 5% salt (NaCl w/w) water as an aversive stimulus. Collado et al. (2021) caught their bees in the wild and brought them back to the lab for testing, while we tested our bees directly in the field. Collado et al. (2021) used larger containers (H x L x W: 130 x 25 × 25 mm) because they included large eusocial bees in their sample, while we used smaller cylindrical containers (L x H: 30.8 x 25.4 mm) because we focused on smaller solitary bee taxa.

### **Stimulus colors**

We measured the reflectance spectra of the two colored paper stimuli used in the current study’s FMPER task were measured in 1 nm increments over the wavelength range 350-700 nm with the Carey 7000 Universal Measurement Spectrophotometer (Agilent Technologies). We used the matplot() function from the pavo package (Maia et al. 2013) to plot the percentage reflectance (Figure S1a). To confirm the colors are perceived differently by bees, we plotted them in hexagonal bee color space (Figure S1b) using the vismodel() and colspace() functions from the pavo package.


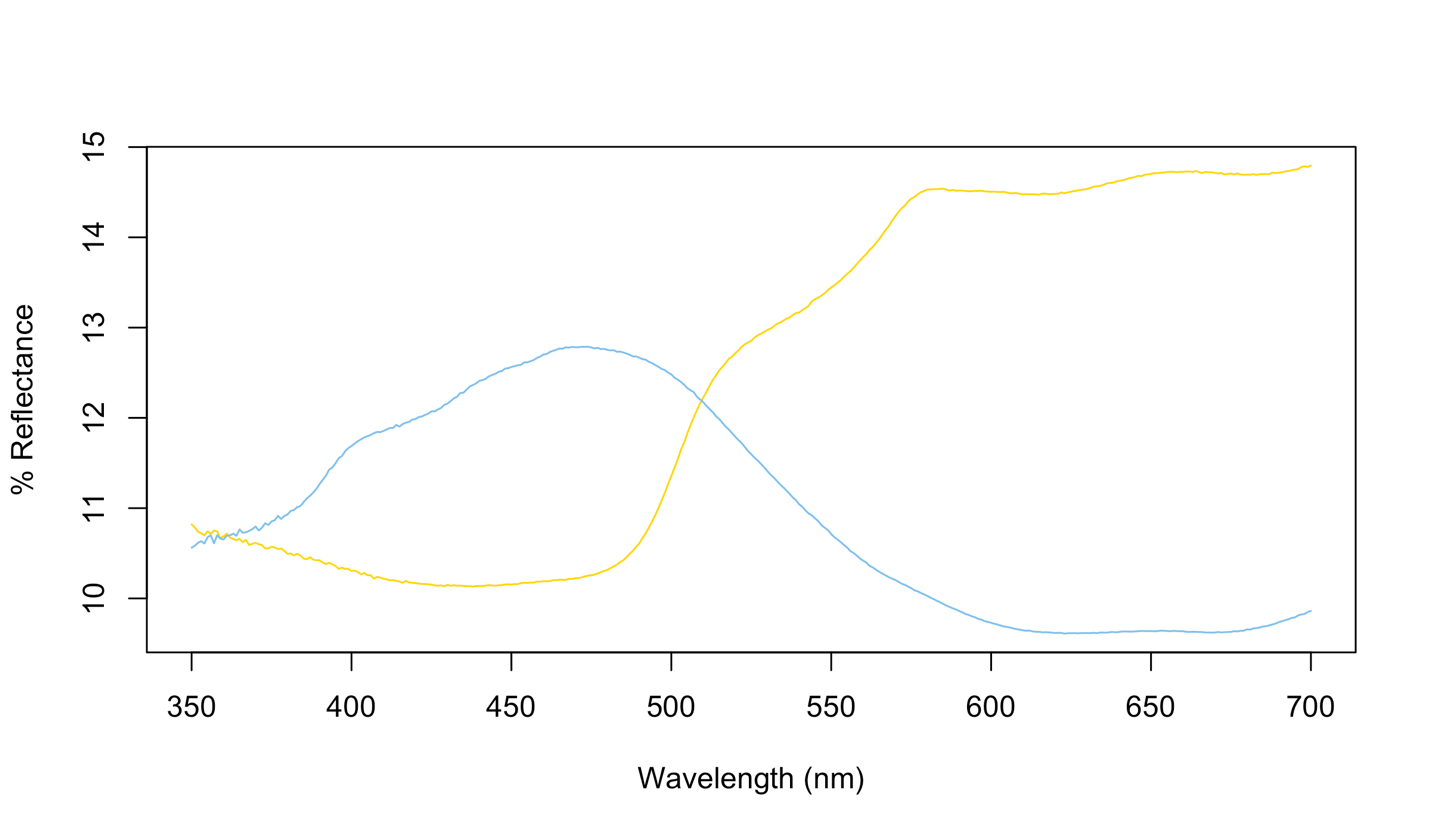


A

**
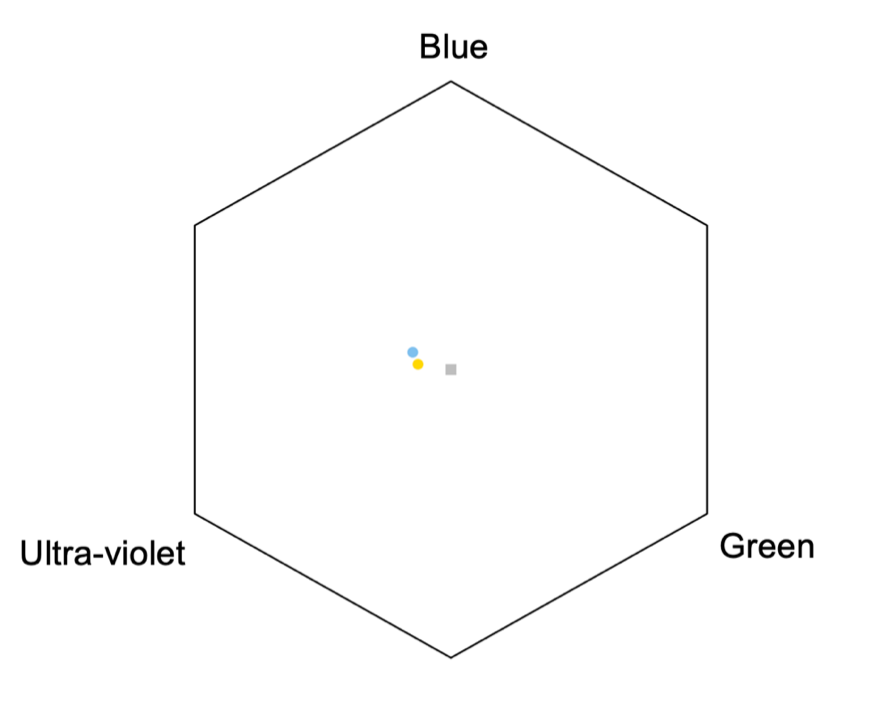
**

B

**Figure S1.** A) Percentage reflectance of the two stimulus colors used in the current study (blue and yellow). B) Bee color space with the two stimulus colors depicted as circles, and a gray square marking the centre of the hexagon. The Euclidean distance between the two color (blue and yellow) loci is 0.046 color hexagon units.

|  | | | | | | | | | | | | | |  |
| --- | --- | --- | --- | --- | --- | --- | --- | --- | --- | --- | --- | --- | --- | --- |
| **Table S1.** List of species tested in the combined dataset with the number of individuals per taxon in the combined dataset, with their corresponding diet breadth, sources for diet breadth, the study for which the bees were tested, the substitutions used in the BeeTree software when the original species could not be used, and the reason for the substitutions. | | | | | | | | | |  |  |  |  |  |
| **Taxa** | **Number of individuals** | **Diet breadth** | **Source for diet breadth** | **Study for which individuals were tested** | | **Substitute in BeeTree** | **Reason for substitute** | | | |  |  |  |  |
| *Agapostemon* spp. | 5 | generalist | No local specialists exist (Fowler and Droege 2020) | Kashetsky et al. (2025) | | *Agapostemon* (*Agapostemon*) *virescens* | Specimens identified to genus level only | | | |  |  |  |  |
| *Andrena* (*Euandrena*) *angustior* | 3 | generalist | Wood & Roberts (2017) | Collado et al. (2021) | |  |  | | | |  |  |  |  |
| *Andrena* (*Melandrena*) *cinerea* | 2 | generalist | Wood & Roberts (2017) | Collado et al. (2021) | |  |  | | | |  |  |  |  |
| *Andrena* (*Opandrena*) *cressonii* | 2 | generalist | Larkin et al. (2008) | Kashetsky et al. (2025) | |  |  | | | |  |  |  |  |
| *Andrena* (*Melandrena*) *dunningi* | 3 | generalist | Johnson (1984) | Kashetsky et al. (2025) | |  |  | | | |  |  |  |  |
| *Andrena* (*Melandrena*) *flavipes* | 6 | generalist | Wood & Roberts (2017) | Collado et al. (2021) | |  |  | | | |  |  |  |  |
| *Andrena* (*Cnemidandrena*) *hirticincta* | 4 | specialist | Larkin et al. (2008) | Kashetsky et al. (2025) | |  |  | | | |  |  |  |  |
| *Andrena* (*Melandrena*) *hispania* (= *A. morio)* | 1 | generalist | Wood (2023) | Collado et al. (2021) | |  |  | | | |  |  |  |  |
| *Andrena* (*Planstandrena*) *pilipes* | 6 | generalist | Wood (2023) | Collado et al. (2021) | |  |  | | | |  |  |  |  |
| *Andrena* (*Callandrena*) *placata* | 1 | specialist | Mitchell (1960) | Kashetsky et al. (2025) | |  |  | | | |  |  |  |  |
| *Andrena* (*Chlorandrena*) *rhyssonota* | 7 | specialist | S. Roberts pers. comm. | Collado et al. (2021) | | *Andrena* (*Chlorandrena*) *humilis* | *Andrena rhyssonota* was not available on BeeTree | | | |  |  |  |  |
| *Andrena* (*Callandrena*) *simplex* | 1 | specialist | Larkin et al. (2008) | Kashetsky et al. (2025) | | *Andrena* (*Callandrena*) *asteris* | | *Andrena simplex* was not available on BeeTree | | | |  |  |  |
| *Andrena* (*Trachandrena*) *spiraeana* | 1 | generalist | Miliczky (1985) | Kashetsky et al. (2025) |  | | | |  | | | | | |
| *Andrena* (*Melandrena*) *vicina* | 2 | generalist | Larkin et al. (2008) | Kashetsky et al. (2025) |  | | | |  | | | | | |
| *Andrena* (*Thysandrena*) *w-scripta* | 1 | generalist | La Berge (1977) | Kashetsky et al. (2025) |  | | | |  | | | | | |
| *Anthophora* (*Lophandrena*) *dispar* | 1 | generalist | Rasmont (1995) | Collado et al. (2021) | *Anthophora* (*Lophandrena*) *pacifica* | | | | *Anthophora dispar* was not available on BeeTree | | | | | |
| *Anthophora* (*Pyganthophora*) *retusa* | 2 | generalist | Mueller & Kuhlmann, (2008) | Collado et al. (2021) |  | | | |  | | | | | |
| *Anthophora* (*Clisodon*) *terminalis* | 1 | generalist | Medler (1964) | Kashetsky et al. (2025) |  | | | |  | | | | | |
| *Augochlora* spp. | 2 | generalist | No local specialists exist (Fowler and Droege 2020) | Kashetsky et al. (2025) | *Augochlora* (*Augochlora*) *pura* | | | | Specimens identified to genus level only | | | | | |
| *Augochlorella* spp. | 2 | generalist | No local specialists exist (Fowler and Droege 2020) | Kashetsky et al. (2025) | *Augochlorella* (*Augochlorella*) *aurata* | | | | Specimens identified to genus level only | | | | | |
| *Augochloropsis* sp. | 1 | generalist | No local specialists exist (Fowler and Droege 2020) | Kashetsky et al. (2025) | *Augochloropsis* (*Paraugochloropsis*) *metallica* | | | | Specimens identified to genus level only | | | | | |
| *Ceratina* spp. | 22 | generalist | No local specialists exist (Fowler and Droege 2020) | Kashetsky et al. (2025) | *Ceratina* (*Zadontomerus*) *calcarata* | | | | Specimens identified to genus level only | | | | | |
| *Colletes simulans* | 2 | specialist | Fowler (2016) | Kashetsky et al. (2025) |  | | | |  | | | | | |
| *Dufourea* *novaeangliae* | 10 | specialist | Lovell & Cockerell (1906) | Kashetsky et al. (2025) |  | | | |  | | | | | |
| *Xenoglossa pruinosa*  (= *Peponapis pruinosa*, *Eucera pruinosa*) | 22 | specialist | Hurd & Linsley (1964) | Kashetsky et al. (2025) | *Eucera* (*Synhalonia*) *acerba* | | | | *Eucera pruinosa* could not be incorporated in the tree due to a BeeTree software issue | | | | | |
| *Eucera* (*Synhalonia*) *rufa* | 1 | generalist | S. Roberts pers. comm. | Collado et al. (2021) | *Eucera* (*Synhalonia*) *edwardsii* | | | | *Eucera rufa* was not available on BeeTree | | | | | |
| *Flavipanurgus venustus* | 3 | specialist | González-Varo et al. (2016) | Collado et al. (2021) |  | | | |  | | | | | |
| *Halictus* spp. | 7 | generalist | No local specialists exist (Fowler and Droege 2020) | Kashetsky et al. (2025) | *Halictus* (*Odontalictus*) *ligatus* | | | | Specimens identified to genus level only | | | | | |
| *Heriades* spp. | 1 | generalist | No local specialists exist (Fowler and Droege 2020) | Kashetsky et al. (2025) | *Heriades* (*Heriades*) *truncorum* | | | | Specimens identified to genus level only | | | | | |
| *Lasioglossum* (*Sphecodogastra*) *immunitum* | 18 | generalist | Ortiz-Sánchez & Pauly (2017) | Collado et al. (2021) |  | | | |  | | | | | |
| *Lasioglossum* (*Sphecodogastra*) *malachurum* | 12 | generalist | Polidori et al. (2010) | Collado et al. (2021) |  | | | |  | | | | | |
| *Lasioglossum* (*Dialictus*) spp. | 17 | generalist | No local specialists exist (Fowler and Droege 2020) | Kashetsky et al. (2025) | *Lasioglossum* (*Dialictus*) *pilosum* | | | | Specimens identified to genus level only | | | | | |
| *Lasioglossum* (*Evylaeus*) spp. | 1 | generalist | No local specialists exist (Fowler and Droege 2020) | Kashetsky et al. (2025) | *Lasioglossum* (*Evylaeus*) *politum* | | | | Specimens identified to genus level only | | | | | |
| *Lasioglossum* (*Hemihalictus*) sp. | 2 | generalist | No local specialists exist (Fowler and Droege 2020) | Kashetsky et al. (2025) | *Lasioglossum* (*Hemihalictus*) *pectorale* | | | | Specimens identified to genus level only | | | | | |
| *Lasioglossum* (*Lasioglossum*) spp. | 2 | generalist | No local specialists exist (Fowler and Droege 2020) | Kashetsky et al. (2025) | *Lasioglossum* (*Lasioglossum*) *coriaceum* | | | | Specimens identified to genus level only | | | | | |
| *Lasioglossum* (*Leuchalictus*) sp. | 1 | generalist | No local specialists exist (Fowler and Droege 2020) | Kashetsky et al. (2025) | *Lasioglossum* (*Leuchalictus*) *leucozonium* | | | | Specimens identified to genus level only | | | | | |
| *Macropis* (*Macropis*) *nuda* | 11 | specialist | Lovell & Cockerell (1906) | Kashetsky et al. (2025) |  | | | |  | | | | | |
| *Megachile* (*Chelostomoides*) *campanulae* | 1 | generalist^†^ | Some sources deem them specialists (Ison et al. 2019; Satyshur et al. 2023) while others consider them generalists (Fowler 2016; Pascarella and Hall) | Kashetsky et al. (2025) |  | | | |  | | | | | |
| *Megachile* (*Xanthosarus*) *gemula* | 1 | generalist | Vizza et al. (2021) | Kashetsky et al. (2025) |  | | | |  | | | | | |
| *Megachile* (*Megachile*) *inermis* | 1 | generalist | Krombein et al. (1979) | Kashetsky et al. (2025) |  | | | |  | | | | | |
| *Megachile* (*Xanthosarus*) *latimanus* | 9 | generalist | Krombein et al. (1979) | Kashetsky et al. (2025) |  | | | |  | | | | | |
| *Megachile* (*Litomegachile*) *mendica* | 1 | generalist | Killewald et al. (2019) | Kashetsky et al. (2025) |  | | | |  | | | | | |
| *Megachile* (*Litomegachile*) *texana* | 1 | generalist | Mitchell (1935) | Kashetsky et al. (2025) |  | | | |  | | | | | |
| *Megachile* (*Xanthosarus*) *willughbiella* | 1 | generalist | Else & Edwards (2018) | Collado et al. (2021) |  | | | |  | | | | | |
| *Melissodes* (*Heliomelissodes*) *desponsus* | 12 | specialist | Lovell & Cockerell (1906) | Kashetsky et al. (2025) |  | | | |  | | | | | |
| *Melissodes* (*Eumelissodes*) *illatus* | 1 | specialist | Lovell & Cockerell (1906) | Kashetsky et al. (2025) |  | | | |  | | | | | |
| *Osmia* (*Helicosmia*) *caerulescens* | 1 | generalist | Else & Edwards (2018) | Collado et al. (2021) |  | | | |  | | | | | |
| *Osmia* (*Helicosmia*) *latreillei* | 4 | specialist | Wafa & El-Berry (1971) | Collado et al. (2021) | *Osmia* (*Helicosmia*) *texana* | | | | *Osmia latreillei* was not available on BeeTree | | | | | |
| *Panurgus* sp. | 1 | specialist | Wood et al. (2022) | Collado et al. (2021) | *Panurgus* (*Panurgus*) *calcaratus* | | | | Specimens identified to genus level only | | | | | |
| *Protandrena* (*Pterosarus*) *andrenoides* | 7 | specialist | Robertson (1926) | Kashetsky et al. (2025) |  | | | |  | | | | | |
| *Rhodanthidium* (*Rhodanthidium*) *sticticum* | 10 | generalist | Muller (1996) | Collado et al. (2021) |  | | | |  | | | | | |
| *Xylocopa* (*Rhysoxylocopa*) *cantabrita* | 1 | generalist | Terzo & Rasmont (2003) | Collado et al. (2021) |  | | | |  | | | | | |
| *Xylocopa* (*Xylocopa*) *violacea* | 2 | generalist | von Reumont et al. (2022) | Collado et al. (2021) |  | | | |  | | | | | |
| ^†^ *Megachile* *campanulae* is considered a generalist with a preference for *Campanula* flowers or a specialist on *Campanula*. Results did not differ with *M.* *campanulae* coded as a generalist or a specialist, thus we report statistics with *M.* *campanulae* as a generalist. | | | | | | | | | | | | |  |  |

**Phylogenetic and diet breadth information**

**
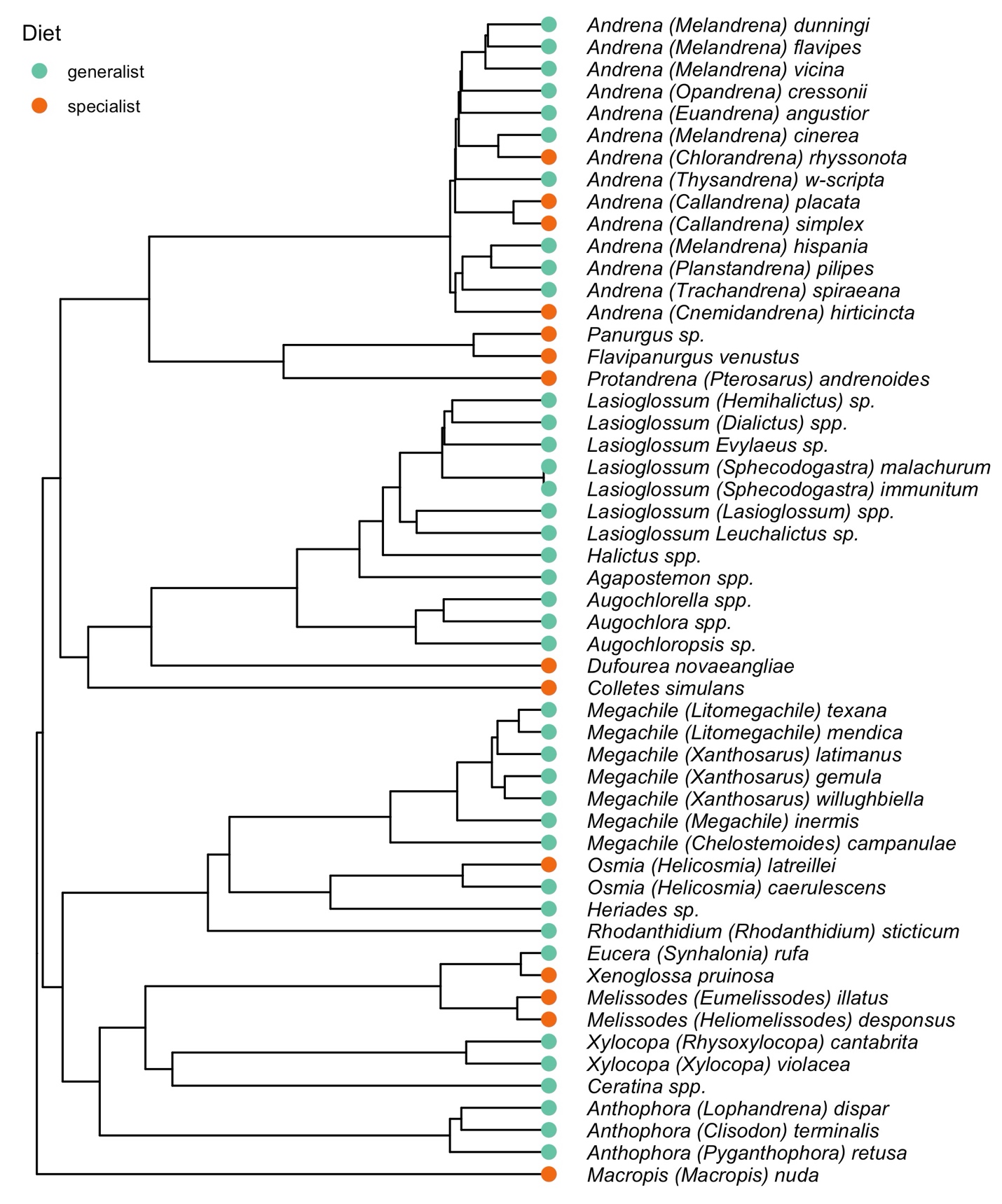
**

**Figure S2.** The phylogenetic tree built using BeeTree (Henríquez-Piskulich et al. 2024), with specialist species indicated with a teal circle, and generalist species indicated with an orange circle.

**
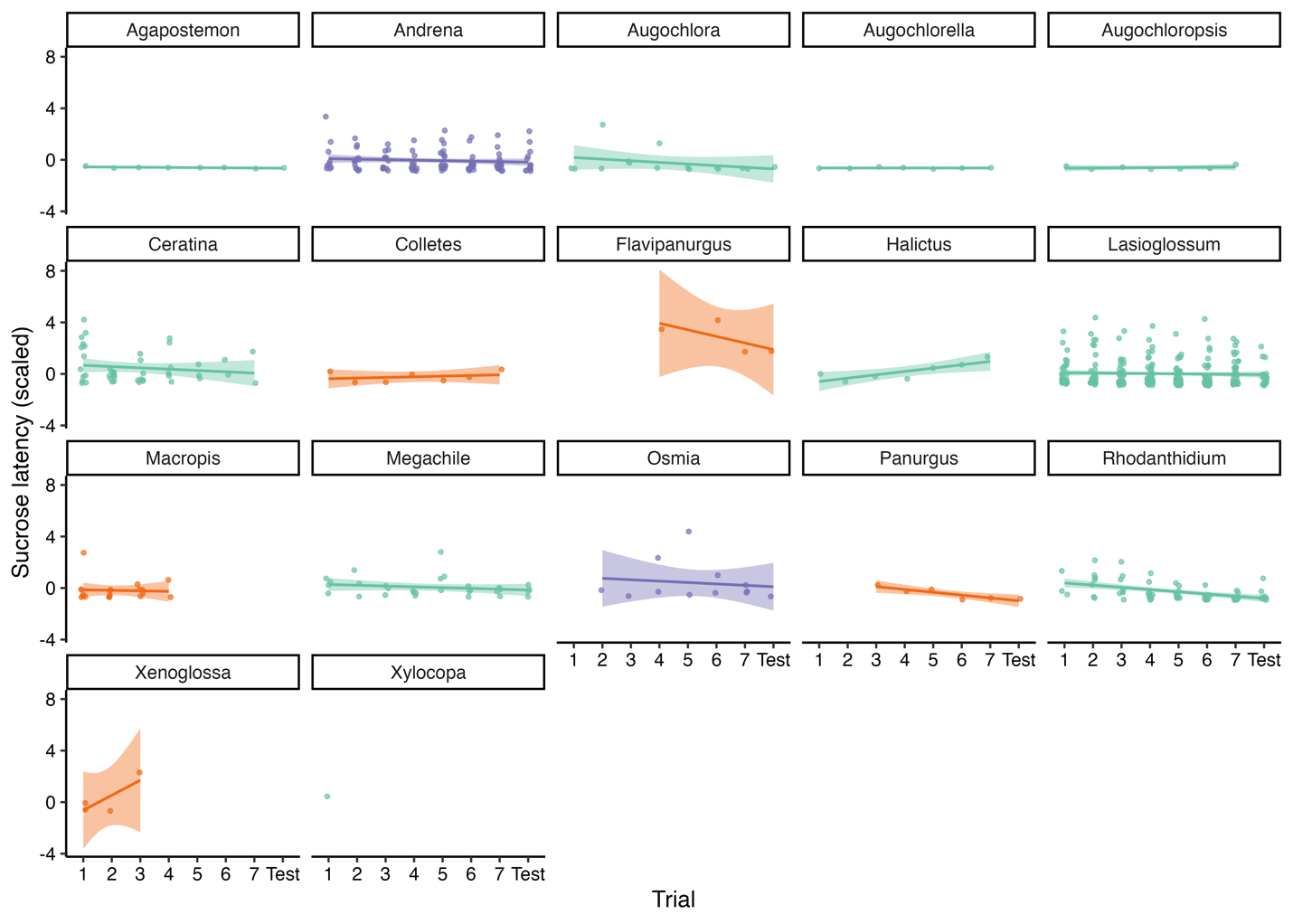
**

**Figure S3.** Sucrose latency as a function of FMPER trial divided into panels for the 17 genera that participated at least once in the combined dataset. Six genera that did not participate, and thus had blank panels, were removed from the figure (*Anthophora*, *Dufourea*, *Heriades*, *Melissodes*, *Protandrena* and *Eucera*). Genera in the combined dataset that include only specialist species appear in orange, genera including only generalist species appear in teal, and genera that include both specialists and generalists appear in purple. Each data point is an individual’s drinking latency for each trial they participated in. The shading is from the geom_smooth() function from ggplot.

**References**

Collado MÁ, Montaner CM, Molina FP, Sol D, Bartomeus I. 2021. Brain size predicts learning abilities in bees. Royal Society Open Science. 8(5):201940. doi:10.1098/rsos.201940.

Else GR, Edwards M. 2018. Handbook of the bees of the British Isles: Volume 1. Ray Society.

Fowler J. 2016. Specialist bees of the Northeast: host plants and habitat conservation. Northeastern Naturalist. 23(2):305–320.

Fowler J, Droege S. 2020. Pollen specialist bees of the eastern United States. Jarrodfowler.com. https://jarrodfowler.com/specialist_bees.html.

González-Varo JP, Ortiz-Sánchez FJ, Vilà M. 2016. Total bee dependence on one flower species despite available congeners of similar floral shape. PLoS One. 11(9):e0163122. doi:10.1371/journal.pone.0163122

Henríquez-Piskulich P, Hugall AF, Stuart-Fox D. 2024. A supermatrix phylogeny of the world’s bees (Hymenoptera: Anthophila). Molecular Phylogenetics and Evolution. 190:107963. doi:10.1016/j.ympev.2023.107963.

Hurd P, Linsley E. 1964. The squash and gourd bees—genera *Peponapis* Robertson and *Xenoglossa* Smith—inhabiting America north of Mexico (Hymenoptera: Apoidea). Hilgardia. 35(15):375–477.

Ison JL, Tuan ES, Koski MH, Whalen JS, Galloway LF. 2019. The role of pollinator preference in the maintenance of pollen colour variation. Annals of Botany. 123(6):951–960. doi:10.1093/aob/mcy211.

Johnson MD. 1984. The pollen preferences of *Andrena* (*Melandrena*) *dunningi* Cockerell (Hymenoptera: Andrenidae). Journal of the Kansas Entomological Society. 57(1):34–43.

Kashetsky T, Raine NE, Forrest J. 2025. Cognitive tasks could be biased towards generalists: a lesson from wild non-eusocial bees. Behavioral Ecology. doi:10.5061/dryad.9cnp5hqx0.

Killewald MF, Rowe LM, Graham KK, Wood TJ, Isaacs R. 2019. Use of nest and pollen resources by leafcutter bees, genus *Megachile* (Hymenoptera: Megachilidae) in central Michigan. The Great Lakes Entomologist. 52(1):8.

Krombein KV, Hurd P, Smith DR, Burks B. 1979. Catalog of Hymenoptera in America north of Mexico. Smithsonian Institution Press Washington, DC.

La Berge WE. 1977. A revision of the bees of the genus *Andrena* of the western hemisphere. Part VIII. Subgenera *Thysandrena, Dasyandrena, Psammandrena, Rhacandrena, Euandrena, Oxyandrena*. Transactions of the American Entomological Society (1890-). 103(1):1–143.

Larkin LL, Neff JL, Simpson BB. 2008. The evolution of a pollen diet: host choice and diet breadth of *Andrena* bees (Hymenoptera: Andrenidae). Apidologie. 39(1):133–145. doi:10.1051/apido:2007064.

Lovell JH, Cockerell TDA. 1906. Notes on the bees of southern maine: Anthophoridae, Halictoididae, Macropidae and Panurgidae. Psyche: a Journal of Entomology. 13(5):109–113.

Maia R, Eliason CM, Bitton P, Doucet SM, Shawkey MD. 2013. pavo: an R package for the analysis, visualization and organization of spectral data. doi:10.1111/2041-210X.12069.

Medler JT. 1964. *Anthophora* (*Clisodon*) *terminalis* Cresson in trap-nests in Wisconsin (Hymenoptera: Anthophoridae). The Canadian Entomologist. 96(10):1332–1336. doi:10.4039/Ent961332-10.

Miliczky ER. 1985. Patterns of resource utilization by spring bees visiting *Salix* with emphasis on use of pollen by bees in the genus *Andrena* (Hymenptera: Apoidea) (competition, resource partitioning). PhD Thesis. University of Illinois.

Mitchell TB. 1935. A revision of the genus *Megachile* in the Nearctic Region. Part II. Morphology of the male sternites and genital armature and the taxonomy of the subgenera *Litomegachile, Neomegachile* and *Cressoniella* (Hymenoptera: Megachilidae). Transactions of the American Entomological Society (1890-). 61(1):1–44.

Mitchell TB. 1960. Bees of the eastern United States. North Carolina Agricultural Experiment Station.

Muller A. 1996. Host‐plant specialization in western palearctic Anthidine bees (Hymenoptera: Apoidea: Megachilidae). Ecological Monographs. 66(2):235–257. doi:10.2307/2963476.

Müller A, Kuhlmann M. 2008. Pollen hosts of western palaearctic bees of the genus *Colletes* (Hymenoptera: Colletidae): the Asteraceae paradox. Biological Journal of the Linnean Society. 95(4):719–733. doi:10.1111/j.1095-8312.2008.01113.x.

Ortiz-Sánchez F, Pauly A. 2017. Contribution à la connaissance des Halictinae d’Espagne, avec un atlas des espèces de la Péninsule Ibérique (Hymenoptera: Apoidea: Halictidae). Belgian Journal of Entomology. 54:1–92.

Pascarella JB, Hall HG. The bees of Florida: *Megachile* - Subgenus *Chelostomoides*. https://entnemdept.ufl.edu/hallg/melitto/Intro.htm.

Polidori C, Rubichi A, Barbieri V, Trombino L, Donegana M. 2010. Floral resources and nesting requirements of the ground‐nesting social bee, *Lasioglossum malachurum* (Hymenoptera: Halictidae), in a Mediterranean semiagricultural landscape. Psyche: a Journal of Entomology. 2010(1):851947. doi:10.1155/2010/851947.

Rasmont P. 1995. Les anthophores de France du sous-genre Lophanthophora Brooks avec la redescription de trois espèces au statut confus (Hymenoptera: Apoidea: Anthophorinae). Vol. 31. Taylor & Francis. p. 3–20.

von Reumont BM, Dutertre S, Koludarov I. 2022. Venom profile of the European carpenter bee *Xylocopa violacea*: evolutionary and applied considerations on its toxin components. Toxicon: X. 14:100117. doi:10.1016/j.toxcx.2022.100117.

Robertson C. 1926. Revised list of oligolectic bees. Ecology. 7(3):378–380.

Satyshur CD, Evans EC, Forsberg BM, Evans TA, Blair R. 2023. Determining Minnesota bee species’ distributions and phenologies with the help of participatory science. PeerJ. 11:e16146. doi:10.7717/peerj.16146.

Terzo M, Rasmont P. 2003. *Xylocopa cantabrita* Lepeletier en France (Hymenoptera, Apoidea). Bulletin de la Société Entomologique de France. 108(5):441–445.

Vizza K, Beresford D, Hung J, Schaefer J, MacIvor S. 2021. Wild bees (Hymenoptera: Apoidea) from remote surveys in northern Ontario and Akimiski Island, Nunavut including four new regional records. The Journal of the Entomological Society of Ontario. 152:57–80.

Wafa A, El-Berry A. 1971. Nesting behaviour of *Osmia latreillei* Spin. and *Osmia submicans* Mor. Bulletin de la Société Entomologique d’Egypte. 55:363–372.

Wood TJ. 2023. The genus Andrena Fabricius, 1775 in the Iberian Peninsula (Hymenoptera, Andrenidae). Journal of Hymenoptera Research. 96:241–484. doi:10.3897/jhr.96.101873.

Wood TJ, Patiny S, Bossert S. 2022. An unexpected new genus of panurgine bees (Hymenoptera, Andrenidae) from Europe discovered after phylogenomic analysis. Journal of Hymenoptera Research. 89:183–210. doi:10.1016/j.ympev.2017.10.007.

Wood TJ, Roberts SPM. 2017. An assessment of historical and contemporary diet breadth in polylectic *Andrena* bee species. Biological Conservation. 215:72–80. doi:10.1016/j.biocon.2017.09.009.
